# Plant developmental oddities

**DOI:** 10.1007/s00425-024-04534-8

**Published:** 2024-09-24

**Authors:** Carlo M. Pozzi, Vittoria F. Brambilla, Angelo Gaiti, Alberto Spada

**Affiliations:** Department of Agricultural and Environmental Sciences, University of Milan, Via Celoria 2, 20133 Milan, Italy

**Keywords:** SAM, Evolutionary adaptation, Plant development, Phytomer, Bauplan

## Abstract

**Main conclusion:**

Plants lacking shoot apical meristem develop with unique body shapes, suggesting rewiring of developmental genes. This loss of the meristem is likely influenced by a combination of environmental factors and evolutionary pressures.

**Abstract:**

This study explores the development of plant bodies in three families (Podostemaceae, Lemnaceae, and Gesneriaceae) where the shoot apical meristem (SAM), a key structure for growth, is absent or altered. The review highlights alternative developmental strategies these plants employ. Also, we considered alternative reproduction in those species, namely through structures like turions, fronds, or modified leaves, bypassing the need for a SAM. Further, we report on studies based on the expression patterns of genes known to be involved in SAM formation and function. Interestingly, these genes are still present but expressed in atypical locations, suggesting a rewiring of developmental networks. Our view on the current literature and knowledge indicates that the loss or reduction of the SAM is driven by a combination of environmental pressures and evolutionary constraints, leading to these unique morphologies. Further research, also building on Next-Generation Sequencing, will be instrumental to explore the genetic basis for these adaptations and how environmental factors influence them.

## Introduction

Structural ontology allows us to approach the vast morphological variability of plants by considering three fundamental, mutually exclusive, plant organ categories: root, stem (caulome), and leaf (phyllome) (planteome.org; Ilic et al. 2007). Despite the usefulness of the ontology approach, plants’ growth occurs in a far less categorized way (Kirchoff et al. 2008).

Due to their peculiar developmental patterns, our search of non-canonical morphologies concentrates on the families Podostemaceae and Gesneriaceae among the dicots and Lemnaceae as monocots*.* Plants in these families show significant developmental alterations compared to model organisms, such as Antirrhinum, Arabidopsis, and maize. In this sense, species of the three families can be considered as “mutants” of the canonical plant *bauplan*, where developmental processes result in unusual morphologies (Sattler 2022).

### The general rules of embryo and shoot development

The plant caulome grows thanks to the activity of the shoot apical meristem (SAM) and, in some species (mostly in monocots, such as the Cyperaceae and Poaceae), also the underlying intercalary meristems (IM), located at the base of internodes. The SAM also provides growth of lateral organs, leading to the development of leaves and, eventually reproductive organs, while at the leaf axils develop axillary meristems (AM) (Kalve et al. 2014), that provide the growth of newly formed lateral branches. The caulome is organized in modules (phytomers) that include, from bottom to top, a bud containing an AM, an internode, and a node with a leaf. Phytomers increase in size and number during plant growth. In model species, at flowering, the SAM enlarges to from an inflorescence meristem. The activity of meristems and the length and organization of phytomers is tightly regulated by genes, hormones, and their interactions (Shi and Vernoux 2022).

**Fig. 1:**
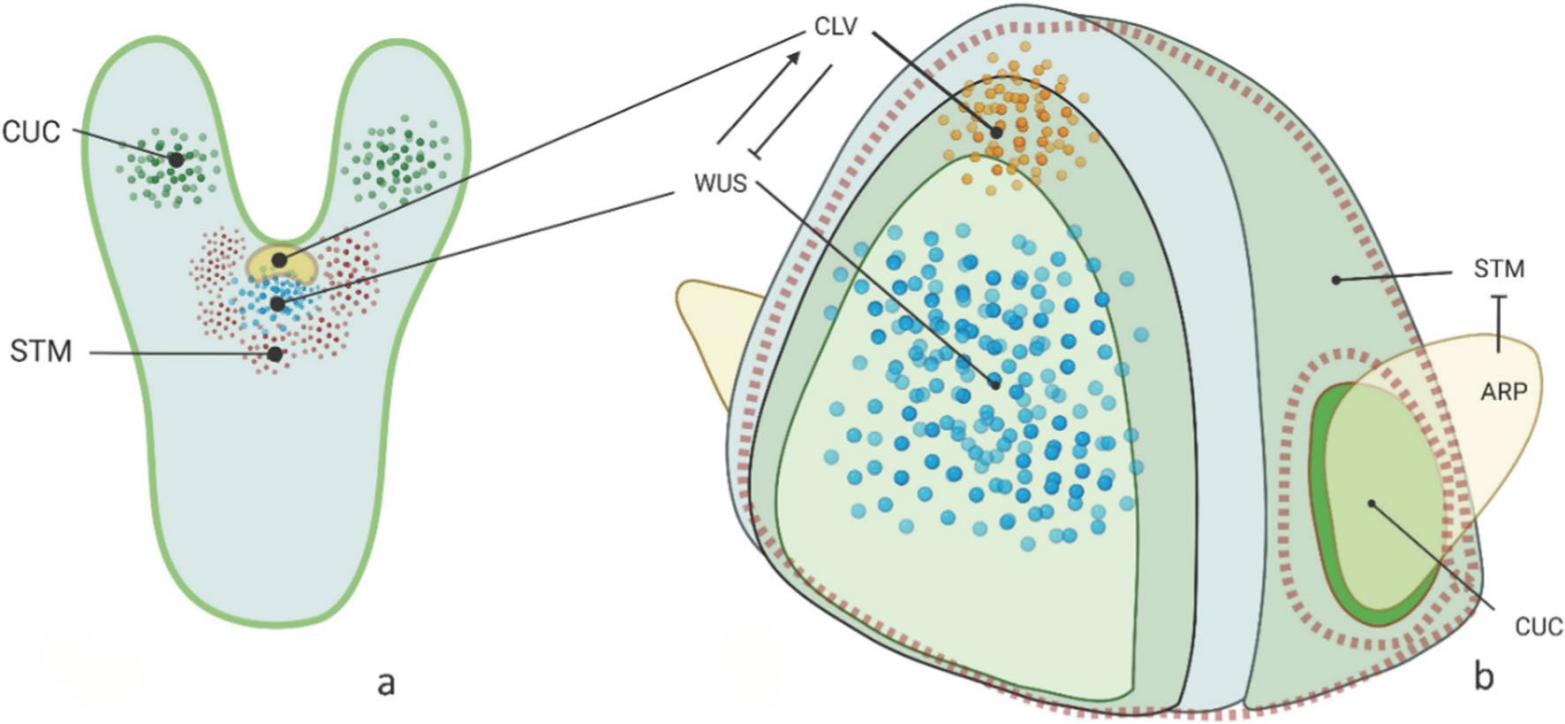
**a** Conceptual model, for a generic dicot species, of a late-heart embryo. **b** Vegetative shoot apical meristem of a model dicot species that has just initiated a leaf primordium as a consequence of the interplay of *STM*, *WUS*, *CLV3*, *ARP*, and *CUC*. *WUS* is transcribed in the OC and activates *CLV3/CLV1*, that in turn inhibits *WUS*. *STM* is expressed in the SAM and is repressed by *ARP* in the leaf primordium. *CUC* marks the boundary between leaf primordium and AM. The layers L1–L3 are indicated by dome-shaped lines. Color code: green dots (**a**) and vivid green area (**b**): *CUC*; red dots (**a**) and area delimited by red hatched line (**b**): *STM*; yellow area (**a**) and yellow dots (**b**): *CLV.* In this context, *CLV* refers to the area where both *CLV3* and *CLV1* are present; blue dots: *WUS*

In Arabidopsis, the SAM is formed in the embryo and originates all the above-ground organs during plant development. The first indication of SAM development, at least in Arabidopsis, occurs around the 16-cell embryo stage, and is characterized by an invariant cell division pattern (Jurgens et al. 1995; Laux et al. 2004; Mansfield and Briarty 1991). As embryogenesis progresses, the SAM becomes more recognizable as a distinct structure with specific cell types and organization, including the cotyledons. Eventually, the embryonic shoot is formed. The SAM (Fig. 1) is characterized by a central zone (CZ), containing the initial (or stem) cells; a peripheral zone (PZ), which generates lateral organs (leaves, flowers), and AM that are formed at the boundary between developing leaves and SAM; a rib zone (RZ) responsible for the proliferation of internal stem tissues. The organizing center (OC) is located below the CZ and regulates cell fate and SAM patterning. The dicots SAM is typically organized into three clonally distinct layers: the surface layer (L1, formed, in Arabidopsis, at the eight-celled embryo stage), which divides forming the epidermis; the L2 subepidermal layer, and the L3, which includes the remaining inner tissues of the shoot (Xue et al. 2020b; Gaillochet et al. 2015). In Arabidopsis, L2 and L3 originate at the heart stage of embryogenesis (Barton and Poethig 1993). The layers are also known as “*tunica*” (the outer layers) and “*corpus*” (the inner mass of cells). The outer L1 layer derives from epidermal precursor cells within the upper tier of the eight-celled proembryo, while L2 and L3 originate at the heart stage of embryogenesis from the subepidermal tissue located in the center of the apical region due to periclinal divisions (Barton and Poethig 1993). In monocots, particularly in the Poaceae, the embryonic stages often show less stereotypic cell division patterns. The zygote undergoes a series of cell divisions transforming it into progressively more complex structures, known as two-celled, quadrant, eight-celled, and dermatogen (Itoh et al. 2005). The dermatogen marks the transition from the early cleaved embryo to the proembryo stage, a structure with defined cell layers and the beginnings of organ development. In grasses, unlike eudicot species, the SAM develops on one side of the embryo during the transition stage (Itoh et al. 2016). This SAM then produces multiple embryonic leaves incorporating some vegetative development before dormancy is established. Noteworthy, monocots such as maize have only one tunica layer (L1) and the inner corpus.

### Genes that determine the primary meristems

Although molecular models that regulate the SAM development derive from studies in model species, the general organization appears to be conserved in numerous other species (Chandler et al. 2008). *WUSCHEL*-related homeobox (*WOX*) transcription factors are crucial for cell fate determination, differentiation, and the regulation of various developmental processes and plant growth, such as the development of lateral and floral organs, balancing embryonic and post-embryonic development, and controlling callus proliferation. (Lau et al. 2012). The *WOX* family is an ancient and conserved group of proteins found from green algae to flowering plants. In club moss, a *WOX* gene is thought to be the ancestor of the *WOX9* and *WUSCHEL* (*WUS*) clades. Across multicellular plants, *WOX* genes are primarily involved in regulating meristem development. The separation of the *WUS* and *WOX5* genes occurred only in the angiosperms, likely due to a gene duplication event in their ancestors. In angiosperms, these genes are expressed in the SAM and RAM, respectively. In Arabidopsis, specific functions of *WOX* genes include the roles of *WUS* and *WOX5* in maintaining stem cell activity in the SAM and RAM, respectively. Additionally, *WOX5* is expressed during the early stages of lateral root and cotyledon development. The *WUS* gene also governs the development of ovules and anthers in Arabidopsis, maize, and rice. Phylogenetic analysis categorizes the *WOX* genes of *Arabidopsis thaliana* into three primary clades, and the most evolutionary recent includes *WUS* and *WOX1*-*7* subfamilies. *WUS*, *WOX4*, and *WOX5* are involved in the regulation of stem cells in the SAM, vascular cambium, and RAM, respectively, while *WOX2* is essential for the proper development of the embryonic apical region. In legumes, *WOX5* also plays a key role in regulating the initiation and development of symbiotic nodules and is a target of the *CLAVATA*-like system, which controls nodulation. It is proposed that the evolution of nodules, a new plant organ resulting from plant–microbe interactions, involved the expansion of *WOX5* role (Lutova et al. 2015). The size of the SAM depends on regulatory elements, such as the mobile peptide encoding *CLAVATA* (comprising the *CLV* peptide *CLV3*, and its receptors *CLV2* and *CLV1*), and the homeodomain gene *WUS*, and on their feedback signaling (Fig. 1a). The *WUS*/*CLV* negative feedback loop maintains a relatively constant pool of stem cells throughout plant life. *WUS* accumulates at a higher level in the rib meristem (located beneath the epidermis in stems and roots) than in the overlying central zone (Snipes et al. 2018). In Arabidopsis, *WUS* expression can be first detected at the globular stage, in small groups of cells of the future apical region. *WUS* protein moves between cells through plasmodesmata into the stem cells, where it induces *CLV3* (Plong et al. 2021). *CLV3* then interacts with the *CLV1* and *CLV2* receptors to inhibit *WUS* in the OC cells, thus controlling meristematic cell identity and stem cells number. The evolution of *CLV3* has played a significant role in the transition from two-dimensional to three-dimensional plant structures. (Agarwal et al. 2022). The *WUS*/*CLV* feedback is further regulated by hormones and hormones–genes crosstalk, where cytokinins maintain meristematic activity while auxins trigger differentiation (Shi and Vernoux 2022; Wang et al. 2018). We mention here two additional genes essential in SAM maintenance and regulation, as modeled in Arabidopsis, which are also studied in the species that we considered: *SHOOT MERISTEM LESS* (*STM*) and *CUP SHAPED COTYLEDONS* (*CUC*). The first is a class-1 KNOX gene, like *BREVIPEDICELLUS* (*BP*), *KNAT2*, and *KNAT6*. *STM* is expressed in the SAM, but not in lateral organ primordia, where it is repressed by *ASYMMETRIC LEAVES1* (a MYB-domain gene of the ARP family; Byrne et al. 2000). *STM* is required during embryogenic SAM development and maintenance, as it keeps the cells in an undifferentiated state. *CUC* belongs to the NAC gene family, and it is expressed at organ or meristem boundaries, contributing to distinguishing the SAM from lateral appendages (Takada et al. 2001). In Arabidopsis, *cuc* mutants have fused cotyledons and lack the SAM (Aida et al. 1997), demonstrating how the apical meristem requires *CUC* expression for proper development (Fig. 1b) (Li et al. 2020). Of the meristem regulatory genes, *WUS* is first expressed at the 16-cell globular stage embryo, in Arabidopsis. At a later stage of embryo development, *CUC* and *STM* genes show overlapping expression in the future cotyledon boundary area. During embryogenesis, *STM* boosts *CUC* expression but also represses *CUC* by indirectly activating microRNA164, which targets *CUC* mRNA. This leads to the formation of a boundary region by inhibiting *CUC* expression where *STM* is present. Consequently, *CUC* expression is excluded from the center of the *STM* region, and *STM* is confined to this central area by the heart embryo stage. The *STM* region eventually forms a domed-shaped SAM, while the growth in the *CUC* region is suppressed, creating a furrow (Nakamura et al. 2024) (Fig. 1b). At the end of the embryogenesis, the *CLV*/*WUS* negative feedback loop coordinates stem cell proliferation and differentiation (Xue et al. 2020b; Zhang et al. 2017).

The regulatory network governing stem cell maintenance in the SAM appears less well defined in monocots compared to dicots. Both groups utilize a negative feedback loop and receptor–ligand complexes in the *CLV/WUS* pathway to regulate the SAM, but differences are observed, as summarized by Kitagawa and Jackson (2019). In rice, the system composed of the *FON1–FON2* genes corresponds to the *WUS/CLV* feedback loop in Arabidopsis, although its function is better characterized in the floral meristem. Furthermore, the functions of some key players, like *WOX4* in rice, seem to diverge from their dicot counterparts (Yasui et al. 2018). Although the functional counterpart of *WUS* has not been clearly identified in maize and rice, the rice *WOX4* functions similarly to *WUS* (Yasui et al. 2018). The rice *WUS* ortholog (known as *TAB1*) is required for SAM initiation more than maintenance (Suzuki et al. 2019). Computational models for maize propose alternative regulatory mechanisms where signals from developing organs might influence stem cell activity in the neighboring SAM (Fletcher 2018).

## Lemnaceae

**Fig. 2:**
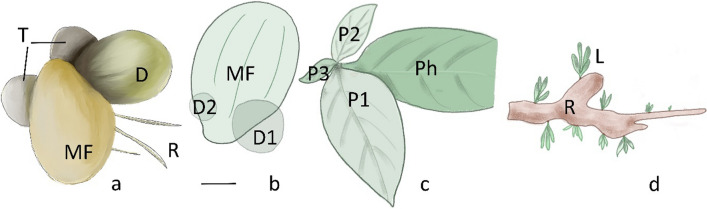
Unorthodox plant morphology of Lemnaceae, Gesneriaceae, and Podostemaceae*.* **a** Aerial view of the body of the *Landoltia* (Lemnaceae), where the dormant vegetative buds (turions) are represented; **b** *Lemna* (Lemnaceae): mother frond with two budding pouches; D1, daughter frond and D2, smaller daughter frond. Note that these tissues are partially covered by the MF; **c** *Streptocarpus* (Gesneriaceae): rosulate plant with cotyledonary phyllomorph (Ph); P1-P3 additional phyllomorphs formed in numbered succession; **d** *Hydrobryum* (Podostemaceae): Ribbon like branching roots (R) with adventitious tufts of leaves on flank (L). *T* turion, aerial view, *MF* mother frond, *DF* daughter frond, arising from the budding pouch, *R* root, *L* leaf. Bar: 1 mm in **a** and **b**, 1 cm in **c**, and 3 mm in **d**

The Lemnaceae (known as duckweeds) is a family of aquatic monocots characterized by an extreme morphological reduction. Species of the family lack the SAM and a proper phytomeric structure, both in the embryo and in the adult plant. They have a “frond” (Hillman 1961), i.e., a dorsiventrally polarized and flattened leaf-like structure, with a variable surface structure depending on the species. Duckweeds reproduce vegetatively by fronds budding, and by “turions”, which are modified, flattened, life-like structure (Fig. 2a). Sexual reproduction via self-pollinating or by entomo-pollinated florets is also possible (Tippery and Les 2020). The family is further divided into Lemnoideae (with a varying number of roots) and Wolffioideae (without roots). The latter is a monophyletic subfamily, while Lemnoideae are paraphyletic. The five genera *Spirodela*, *Landoltia*, *Lemna*, *Wolffia*, and *Wolffiella* (Bog et al. 2020) can be distinguished based on vegetative features: large fronds with multiple roots (*Landoltia* and *Spirodela*); fronds with one root each (*Lemna*); small fronds with no roots (*Wolffia*); small, elongated fronds and no roots (*Wolffiella*). The duckweeds are reported to be closely related to the Araceae (Les and Crawford 1999; Les et al. 1997; Tippery and Les 2020). It is hypothesized that evolution in the family proceeded from complex to reduced forms, i.e., from the largest and more complex genus *Spirodela* to the smaller and simpler genus *Wolffia* (Landolt 1986; Les et al. 1997).

### Plant morphology

During embryogenesis of *Lemna paucicostata*, the first frond originates from a proembryo mound derived from one or two cells located below the epidermis (Maheshwari and Kapil 1963), and it is wrapped by a cotyledonary sheath. A short, few-celled pedicel (stipe) connects the frond to the embryo’s body. Shortly after the first frond attains a size of about 20 cells, a protuberance arises at the junction of the pedicel and the laminar portion of the frond, a structure known as “daughter frond”, which undergoes the same development as the “mother” (Fig. 2a, b; Fig. 7 Lemnaceae). Importantly, the frond formation does not involve any active SAM.

The shoot of *Spirodela polyrhiza*, *Lemna minor,* and *Wolffia borealis* is characterized by the production, in the pocket(s) of older fronds, of daughter fronds that eventually separate (Yang et al. 2021; Lemon and Posluszny 2000).In the smaller *W. borealis,* one pouch only appears where daughter fronds originate and develop. The meristematic-like cells of the new frond are at the base of the previously formed daughter frond.In *S. polyrhiza*, fronds at maturity are 4 to 8 mm in length. The outer edge of the frond develops into a prophyll, a collar of cells surrounding the new originating “bud”.

The characterization of frond and flower development in *L. aequinoctialis* (Yoshida et al. 2021) shows that no evident SAM is present during frond formation. However, staining with ethynyl deoxyuridine during frond development indicates the existence of a meristematic-like region, proximally located in the daughter frond (Yoshida et al. 2021).

### Genetics and genomics

The genome of several species of Lemnaceae has been sequenced: *Spirodela polyrhiza* (Michael et al. 2017); *Spirodela intermedia* (Hoang et al. 2020); *Lemna minor* (Van Hoeck et al. 2015); *Wolffia australiana* (Michael et al. 2020), and *Lemna minuta* (Abramson et al. 2022)*.* The genome size varies from 150 (*S. polyrhiza*) to 1,881 Mb (*W. arrhiza*) (Hoang et al. 2022). The number of non-redundant gene coding proteins is relatively low (from 15,000 in *W. australiana* to 19,000 in *S. polyrhiza*), and the number of genes in gene families is reduced. For example, there are about 150 genes for lignin biosynthesis in *Arabidopsis thaliana* and just about 70 in *Spirodela* (An et al. 2018; Park et al. 2021; Lam and Michael 2022; Michael et al. 2020). In *Wolffia*, the Benchmarking Universal Single-copy Orthologs (BUSCO) set of genes is reduced: apparently several hundred genes involved in root development, light signaling, terpene biosynthesis, and innate immunity pathways have been lost during evolution (Michael et al. 2020; Lam and Michael 2022). Genes in the sphingolipid pathways, on the other hand, are overrepresented. This may happen, because sphingolipids are known contributors to the maintenance of the structure and stability of cell membrane and to the plant response to abiotic stresses, two roles important for species—such as the Lemnaceae—fast growing in aquatic environment. In addition, the genus *Wolffia* is missing several gene families of the small signal peptide *CLV3*/*ESR-RELATED* group (Michael et al. 2020). Despite gene losses, *Wolffia* maintains a set of gene families shared with plants characterized by a minimal size expansion (Michael et al. 2020). In *Lemna minuta*, using single-nuclei transcriptome and a chromosome-resolved genome, distinct cell types representing meristem, the leaf-stem fusion (frond), and root-like tissues (Abramson et al. 2021) have been identified. Transcriptomic analysis on turions and fronds of *S. polyrhiza* revealed that genes involved in stress tolerance as well as starch and lipid metabolisms are differentially expressed in the two tissues. Probably, the turion function as dormant bud is due to the reprogramming of key regulatory genes (among which the Growth-Regulating Factors), which also control seed maturation and germination (Pasaribu et al. 2023; Li et al. 2022).

## Podostemaceae

The family belongs to the Malpighiales, with Hypericaceae as its sister family (Ruhfel et al. 2011). Podostemaceae are also known as river-weeds. In tropical and sub-tropical rivers and waterfalls, their vegetative part is a dorsiventrally flattened photosynthetic body that adheres to the hard, rocky, substrate. They survive the monsoon as submerged haptophytes and rheophytes. The body is either described as creeping roots (“root crusts”) or creeping stems (“shoot crusts”), depending on species or subfamily (Rutishauser 1997). The plants flower and fruit during the dry season, and their seeds germinate underwater (Koi and Kato 2010). The family includes about 280 species and 50 genera, 26 being monospecific and most with less than 10 species. The family is subdivided into three subfamilies Podostemoideae, Weddellinoideae, and Tristichoideae. The Podostemoideae comprise paraphyletic American clades, a monophyletic Madagascan, and a monophyletic Asian clade, where the genus *Cladopus* and *Hydrobryum* form a subclade, while *Zeylanidium* is separate (Koi et al. 2012). Only one species, *Weddellina squamulosa,* is present in the Weddellinoideae. Distinctive characters are the floral and capsule structures: three carpels (capsule valves) in the Tristichoideae, two carpels in the other two groups. In the three subfamilies, the symmetry of flowers and roots is variable (Koi et al. 2015).

### Plant morphology

**Fig. 3:**
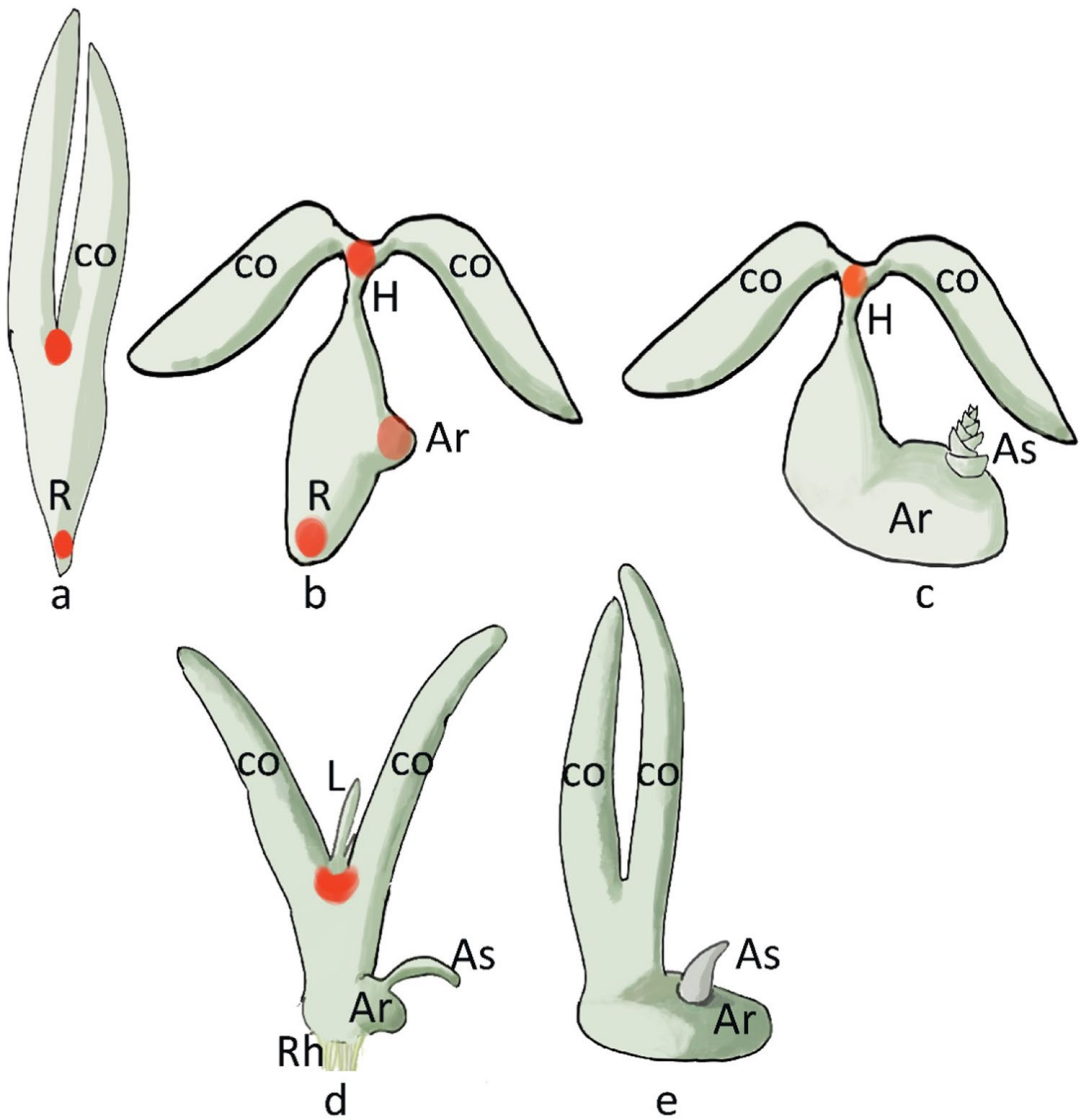
Comparative schematic representation of the early body plan of different plants. **a** *A. thaliana*; **b** Tristichoideae; **c** Weddellinoideae; **d** Podostemoideae (*Zeylanidium*); **e** Podostemoideae (*Hydrobryum*). *co* cotyledon, *R* root; *AR* adventitious root, *AS* adventitious shoot, *L* leaf-like structure, *Rh* rhizoid, *H* hypocotyl, *RAM and SAM* red circle. The partially functional pseudo-SAM of *Zeylanidium* is represented by a red crescent. The AS is indeterminate in **c** and determinate in **d** and **e** (lacking SAM)

This family is characterized by a large amount of morphological variation, as a response to adaptation to aquatic environments (Fig. 3b–e). The root (when present) can vary from sub-cylindrical to flattened and foliose. In the Podostemaceae, the leaves and stems are usually replaced by a unique structure, called “thallus”, which is flattened and/or ribbon-like, and anchors the plant to the rocks (Kita and Kato 2005; Koi and Kato 2010). Shoots vary in size, structure of the thallus, and branching; they typically arise proximal to the root near the meristem. In the Podostemoideae, determinate shoots arise near the root tip and flowers are ectopically initiated inside the stem (*Ledermanniella letouzeyi*) (Schenk et al. 2015). Plants of the family lack double fertilization and have no endosperm (Sehgal et al. 2011). In the subfamilies, the origin of organs can be different: Tristichoideae and Weddellinoideae produce leaves-like organs from the SAM, and the plumule (primary shoot) forms between the cotyledons; Podostemoideae have very reduced or no shoots (Fujinami et al. 2013; Fujinami and Imaichi 2015; Imaichi et al. 2005, 1999; Jäger-Zürn 1997; Katayama et al. 2010; Koi et al. 2005, 2012; Koi and Kato 2007, 2010; Rutishauser and Huber 1991; Suzuki et al. 2002). In the majority of Podostemoideae, such as in *Zeylanidium lichenoides* and in the *Cladopus* clade, multiple leaves emerge between the cotyledons in the absence of a distinct shoot meristem, although a potential OC may be present at the 16-cell stage embryo (Katayama et al. 2011). From this peculiar meristem, some leaves arise during germination. In other species (e.g., in the clade *Hydrobryum japonicum),* neither SAM nor leaves are formed (Kita and Kato 2005; Koi and Kato 2007; Suzuki et al. 2002).

**Fig. 4:**
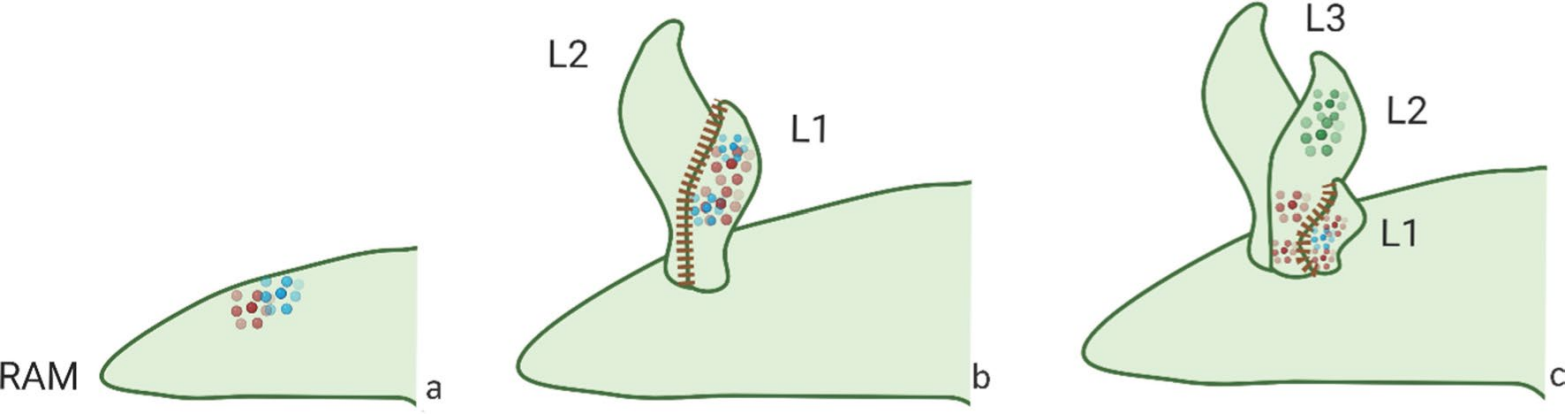
Schematic representation of gene expression in the root of *Hydrobryum japonicum*. **a** *WUS* (blue dots) and *STM* (red dots) are expressed in the root at the site of formation of the first leaf primordium; **b** *STM* and *WUS* are expressed during the initiation and formation of a leaf; **c** A new leaf primordium arises at the base of the previous one, where both *WUS* and *STM* are expressed. *L1* youngest leaf, *L2* leaf following L1, *L3* oldest leaf. Red dots: *STM*; blue dots: *WUS*; green dots: *ARP*; dashed line: abscission-like vacuolated cells

In the Tristichoideae*,* an SAM is present at the end of the embryogenesis, and a primary root originates at the end of the hypocotyl (for example, in *Indotristicha,* and *Terniopsis*). Some genera (*Tristicha*, *Terniopsis*) have short-lived shoots (“*ramuli*”) with three rows of scale-like leaves, an apical meristem, and adventitious shoots deriving from roots; in *Indotristicha,* ribbon-like adhesive roots and root-borne branched shoots are present. Other genera of Tristichoideae (e.g., *Dalziella*) lack roots and their foliose or flattened shoots originate from the fusion of various axes (Rutishauser 2016) (Fig. 3b). In Weddellinoideae and Podostemoideae, primary root and root apical meristem (RAM) are absent, and adventitious roots are formed from the hypocotyl (Kita and Kato 2005; Mohan Ram and Sehgal 1997; Suzuki et al. 2002). In *W. squamulosa,* an SAM is present between the cotyledons and develops into lateral organs and branches, and smaller shoots develop from roots (Koi and Kato 2007) (Fig. 3c). In the Podostemoideae, the seedlings typically lack an embryonic shoot meristem. In many species (e.g., *Zeylanidium lichenoides, Cladopus*), the embryonic shoot is determinate and forms several plumular leaves (i.e., first true leaves that emerge from a germinating seed; Fig. 3d). In other species, the SAM is absent as well as the plumular leaves (e.g., *Hydrobryum japonicum*; Fig. 3e) (Mohan Ram and Sehgal 1997; Sehgal et al. 2007; Suzuki et al. 2002); the plant body is an adventitious root arising from the lateral side of the hypocotyl, progressively forming adventitious shoots where each leaf develops from the base of the opposite second young leaf, in a position where groups of dividing cells are located. The adventitious shoots regularly repeat a process of leaf formation, where each new leaf forms at the base of an existing leaf. The two leaves separate when vacuolated cells in between them detach from the surrounding tissue (Sehgal et al. 2002) (Fig. 2d; Fig. 4b, c).

In the Tristichoideae, an evolutionary trend is observed, that leads to foliose shoots (in *Dalzellia*, for example), from the fusion of the lateral branches which are growing independently in other Tristichoideae (Fujinami and Imaichi 2015). The Podostemaceae evolved a unique body plan with a reduced shoot system and horizontal root system. This adaptation, likely due to novel shoot organogenesis, allows them to colonize submerged rock surfaces under high water pressure.

### Genetics and genomics

The expression of the orthologs of *STM*, *WUS*, *ARP*, and *CUC* have been characterized in the shoot of Tristichoideae and in the SAM-less shoots of the Podostemoideae. In Tristichoideae, the orthologous regulatory genes expression corresponds to that of model plants (Fujinami and Imaichi 2015). In the Tristichoideae *Terniopsis minor, Dalziella ubonensis*, and *Indodalzellia gracilis*, *STM* is located close to the root apical meristem in the epidermal layer and inner tissue of the incoming primordium. Later, in development, *STM* is expressed in the adventitious shoot forming on the root, but absent in leaf primordium tissues developing on its flank. The *WUS* ortholog is expressed in the inner tissue of the primordium, at the point where the shoot develops from the root. At maturity, *WUS* is active in SAM inner cells, but absent in the epidermis and leaf primordium (Fujinami and Imaichi 2015).

In *Hydrobryum japonicum* (Podostemoideae) (Fig. 4), *STM* and *WUS* are expressed in the leaf primordium of young vegetative shoots, proximal to the RAM, and later in the developing new leaf, at the base of the older ones. During development, *WUS* disappears, *STM* becomes restricted to the shoot base, probably due to its pseudo-meristematic nature, and *ARP* is localized distally. *WUS* is expressed in the center of leaf primordia, while the *AS1* ortholog complements *STM* expression in developing vegetative leaf primordia and is expressed distally in the developing leaf. Shifts in gene expression for SAM maintenance and leaf initiation likely cause leaves to emerge from the shoot tip (apex) instead of the periphery. The origin of adventitious shoots near the root meristem may be due to *WUS* ectopic expression, similar to what is observed in Arabidopsis (Katayama et al. 2010; Nakayama 2024). In *Cladopus japonica,* only *STM* has been investigated in vegetative shoots, and found to be expressed in the entire leaf primordium, later confined at the base where a new leaf primordium originates (Katayama et al. 2010).

**Fig. 5:**
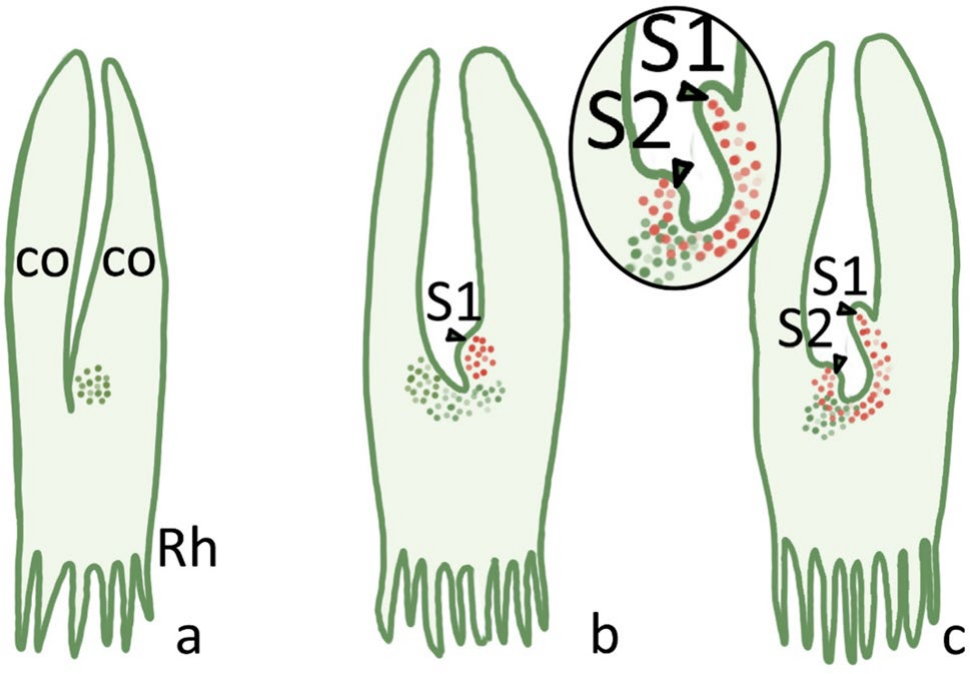
Schematic illustration of gene expression pattern during the early development of *Zeylanidium tailychenoides*. **a** *CUC3* expression (green) at the base of the cotyledon at the onset of a new shoot-like structure; **b** *STM* expression (red) at the site where the new shoot develops (S1); *CUC3* expression (green) where the next shoot primordium (S2) is originating; **c** *STM* (red) expression at S1 and S2. *co* cotyledon, *Rh* rhizoid. The inset provides the details of the pattern of gene expression

In *Zeylanidium tailychenoides* (Fig. 5), the expression of *STM* and *CUC3* has been investigated from embryogenesis to seedling development. Similarly to model species, *STM* transcripts are at first present in the OC precursors of the 16-celled embryo and in a few cells at the fork of the emerging cotyledons (in junction cells). The first two leaves arise from a cryptic meristem present at the base of the cotyledons. *CUC3* is expressed in adaxial epidermal cells at the base of the cotyledons, particularly where the first meristematic region initiates (Fig. 5a). *STM* expression expands between emerging leaf primordia, at the base of the two cotyledons, leading to an asymmetric development of subsequent leaves (Fig. 5b), and its expression domain enlarges when the leaf primordium protrudes (Katayama et al. 2013; Fig. 5c). In the newly developing shoots, *CUC3* and *STM* are expressed in an almost mutually exclusive way (Katayama et al. 2019; Fig. 5c).

### Genetics and genomics

For Podostemaceae, the genome of *Cladopus chinensis* (Podostemoideae) (Xue et al. 2020a) is available. It consists of 827.92 Mbp, with N50 of 142 Mbp, and 27,370 annotated protein-coding genes. Members of gene families involved in shoot development have been annotated, but their expression is not studied. In this species, the available transcriptome allows the identification of genes putatively involved in the establishment of the SAM. More than 600 shoot expressed genes have been identified, some of which are involved in the biosynthesis of hormones, and some are homologous to transcription factors belonging to the *MYB*, *bHLH*, and *HD-zip* families. Gene expression variability exists for specific members of gene families involved in SAM definition, for example the homologs of *ASYMMETRIC LEAVES* (*CcASL1*, *CcASL2*, *CcASL7*, *CcASL8*) and *SHOOT MERISTEMLESS* (*CcSTM1*) are significantly expressed in shoot tissues, while others (*CcWUS10*, *CcASL4*, *CcASL9*, *CcSTM3*, and *CcSTM4*) are expressed at lower levels (Xue et al. 2020b). The functional meaning of those differences remains to be interpreted.

## Gesneriaceae

The Gesneriaceae family includes 150 genera, and 3,400 species of perennial herbs, small trees, or shrubs present particularly in the tropics (Weber et al. 2013). The family includes the sister subfamilies Sanangoideae and Gesneroideae, almost exclusively distributed in the new world, and the Didymocarpoideae, species mainly restricted to the old world (Ogutcen et al. 2021). In Gesneroideae, the enlargement of cotyledons after germination is uniform, while it is uneven in the Didymocarpoideae (Burtt 1963), which show anisocotyledony (i.e. the unequal development of cotyledons) and the presence of endosperm in the seed (Smith 1996). The Didymocarpoideae include the genus *Streptocarpus* (with about 130 species from Africa, Madagascar, and Comoro Islands) and *Monophyllaea* (Mayer et al. 2003). Phylogenetic analyses show that the peculiar morphology of the family evolved independently leading to at least two distinct lineages (Ayano et al. 2005; Ishikawa et al. 2017).

### Plant morphology

Out of the 85 genera of the Didymocapoideae subfamily, only two (*Streptocarpus* and *Monophyllaea*) have an abnormal morphology. Moreover, *Streptocarpus* can be divided into two major clades, one broadly corresponding to the caulescent (i.e. showing a developed stem) group (with conventional shoot architecture; subgenus *Streptocarpella*) and the other mainly composed of acaulescent species (subgenus *Streptocarpus*). The caulescent species form stems and leave from an SAM (Nishii et al. 2015). A phylogenetic analysis suggests that growth forms (caulescent and acaulescent) have multiple origins in this genus (Möller and Cronk 2001).

### Genus *Streptocarpus*

**Fig. 6:**
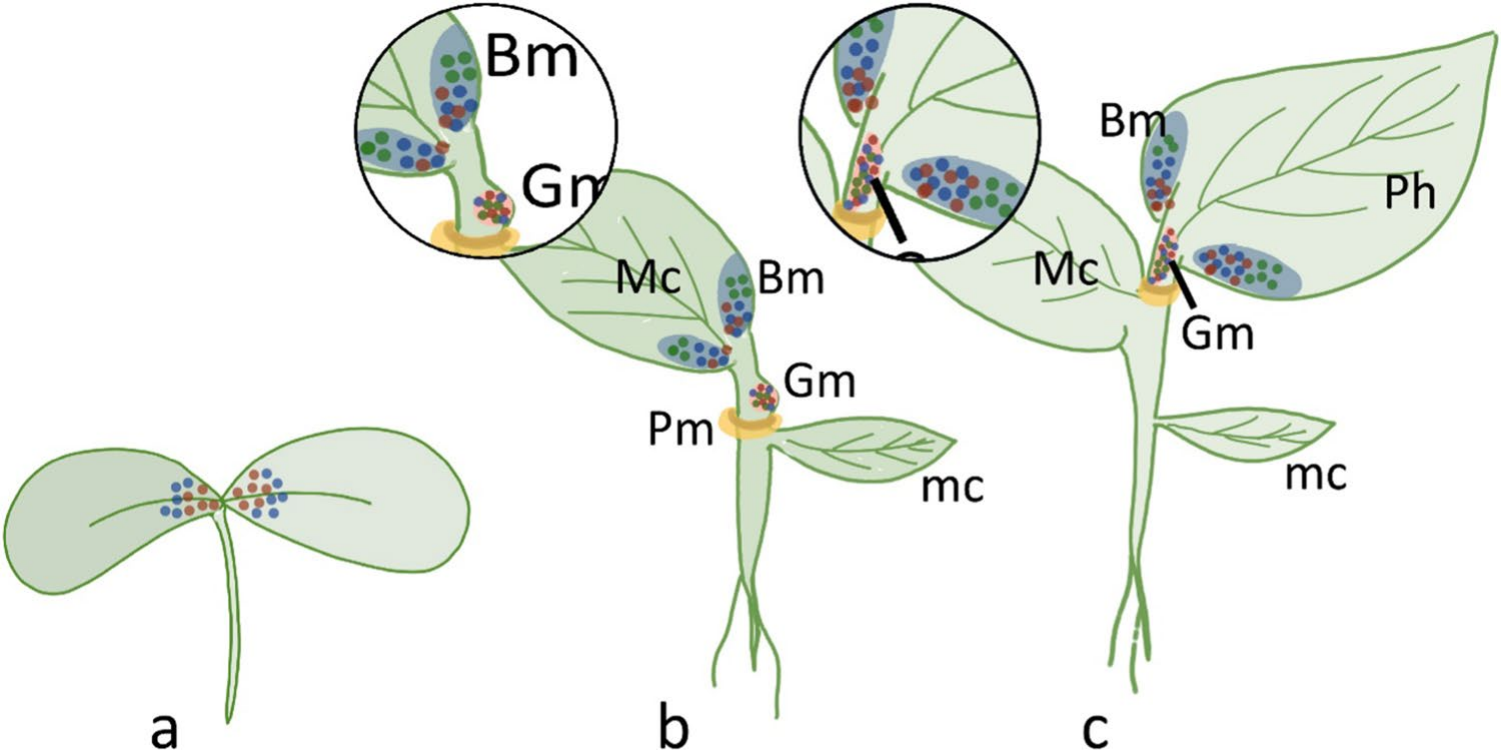
Schematic representation of the formation of micro- (mc) and macrocotyledons (Mc) in *Streptocarpus rexii*. **a** Isocotiledonary early stage; **b** Anisocotyledonary stage; **c** formation of the first phyllomorph. The figure also reports the gene expression patterns in seedlings of *Streptocarpus rexii*. **a** The orthologs of *STM* and *WUS* are expressed in both cotyledons during early stage of germination; **b** *STM*, *WUS*, and *ARP* transcripts are present in the basal meristem (Bm) of the macrocotyledon (Mc); **c** later stage of development, where *STM*, *WUS*, and *ARP* are expressed in the Bm of the phyllomorph (Ph) and in the groove meristem (Gm). *Bm* basal meristem, *Pm* petiolode meristem, *Gm* groove meristem, *co* microcotyledon. Color code: red dots, *STM* expression; blue dots, *WUS*; green dots, *ARP*; blue area: Bm; light red area: Gm; yellow area: Pm. The insets provide details of the pattern of genes expression

The two *Streptocarpus* clades are classified also based on phylogeny and chromosome number (Möller and Cronk 2001; Burtt and Hilliard 1971). The caulescent species of clade I have 15 chromosomes and are caulescent. Clade II species (subgenus *Streptocarpus*) have 16 chromosomes and lack a vegetative SAM and, consequently, a stem. Some acaulescent unifoliate specieshave a single leafy organ that arises by continued growth of one cotyledon (Imaichi 2000). In rosulate species, additional leafy organs are produced from existing ones in the absence of a conventional SAM, forming a sort of rosette (for example, in *Streptocarpus rexii)* (Harrison et al. 2005; Kinoshita and Tsukaya 2019). *Streptocarpus* embryos lack the SAM and, upon emergence, have two isocotyledons and a hypocotyl (Fig. 6a). Later, in development, a cotyledon, indicated as macrocotyledon, becomes larger than the other (Fig. 6b). Subsequently, a cotyledonary petiole arises and, together with the lamina, it constitutes the cotyledonary phyllomorph, a composite structure that combines features of both a leaf (lamina) and a stem (petiolode). New phyllomorphs arise on the petiole near the base of the lamina (Fig. 2c) (Jong and Burtt 1975). The development of the phyllomorph is controlled by three meristems at the junction of laminapetiolode: the basal meristem (Bm), which provides the growth of the lamina; the petiolode meristem (Pm), involved in the growth of the midrib and the elongation of the petiolode; the groove meristem (Gm), which provides the growth of new phyllomorphs and/or inflorescences (Jong and Burtt 1975; Mantegazza et al. 2007) (Fig. 6b, c). As a sign of potential meristematic activity, cell division occurs in the proximal regions of the isocotyledons (Nishii and Nagata 2007). Later, cell divisions occur only at the proximal end of the macrocotyledon. Cell division is also observed at the petiolode, where Gm arises with a tunica-corpus like structure. The Bm is present in all Gesneriaceae (Didymocarpoideae), but its activity is most extended in *Streptocarpus* and *Monophyllaea*, showing that anisocotyly is a prerequisite for the phyllomorph evolution, even if it occurred several times independently and in different environments (Africa and Madagascar) (Nishii et al. 2017).

In unifoliate *Streptocarpus* species (Imaichi 2000), the macrocotyledons become the only phyllomorph, while GM differentiates into an inflorescence (instead of producing new phyllomorphs).

### Genus *Monophyllaea*

In this genus, new organs are not formed after germination until the appearance of the inflorescence (Ayano et al. 2005). Embryos and germinated seedlings of *Monophyllaea* contain two cotyledons of equal size and the SAM is absent. Soon after germination, a meristem-like cluster of small cells appears at the base of each cotyledon. This region disappears on one of the two cotyledons, and the other develops into a macrocotyledon, supported by the Bm (Tsukaya 1997). The Gm, arising at the anisocotyledon stage, produces inflorescences (Ayano et al. 2005).

### Genetics and genomics

The expression of the orthologs of *STM*, *WUS*, and *ARP* have been studied in both *Streptocarpus* and *Monophyllaea* (Nishii et al. 2017). In *S. rexii,* embryogenesis *STM* is not present between the two cotyledons (Fig. 6a). At germination, *STM* expression is restricted to the base of both cotyledons, but later it is noted only for the macrocotyledon (Fig. 6b) (Mantegazza et al. 2007), and subsequently in GM and petiolode. During embryogenesis, *WUS* expression is present in both cotyledons but not between them. Later, it is restricted to the base of the macrocotyledon and in Bm and Gm when it appears on the petiolode (Fig. 6c) (Mantegazza et al. 2009). During the subsequent seedling development, *WUS* is detected in the basal part of the first phyllomorph. *ARP* is expressed in both caulescent *Streptocarpus glandulosissimus* and acaulescent *Streptocarpus rexii* (Fig. 6c) (Nishii et al. 2010). The *ARP* ortholog transcripts are present in seedlings at the anisocotyledon stage in the proximal region of the macrocotyledon, where the Bm is expected to form. This gene is also expressed in the developing lamina of the newly formed phyllomorph, and throughout Bm and Gm. A similar pattern is observed for the *BP* ortholog, and this is confirmed in *S. glandulosissimus* (Nishii et al. 2010). During the evolutionary divergence of the Gesneriaceae family, the Didymocarpinae lineage might have gained the characteristic of anisocotyly. This could have coincided with an expansion of *STM* expression from the SAM to the cotyledons. Furthermore, the expression of KNOX genes in the basal meristem remains indeterminate only in the unifoliate (single-cotyledon) *Streptocarpus* species (Nishii et al. 2017). The orthologs of the *YABBY* genes *GRAMINIFOLIA* (*GRAM*), and *FILAMENTOUS FLOWER* (*FIL*) are involved in organ polarity, promoting lamina growth in *S. rexii* (Tononi et al. 2010). During seedling development, *GRAM* expression is associated with the BM of the macrocotyledon and phyllomorphs, but not with GM, suggesting that an altered regulation of the *GRAM* ortholog may underlay the evolution of the basal meristem. In *Monophyllaea glabra*, *CUC* and *STM* expression overlaps from embryogenesis until the start of the reproductive phase, unlike in *Arabidopsis thaliana*, where these genes become separately expressed in the mature SAM (Nakamura et al. 2024)*.*

Next-Generation Sequencing transcriptome is available for *S. rexii* (Chiara et al. 2013). A genetic map of the species is available based on SNP markers obtained by RAD sequencing (Chen et al. 2018). The first highly contiguous genome of *S. rexii* was published in 2022: it is composed of 5,855 scaffolds covering 766 Mb (83% of the genome), derived from long-read sequences (Nishii et al. 2022).

## Discussion

**Fig. 7:**
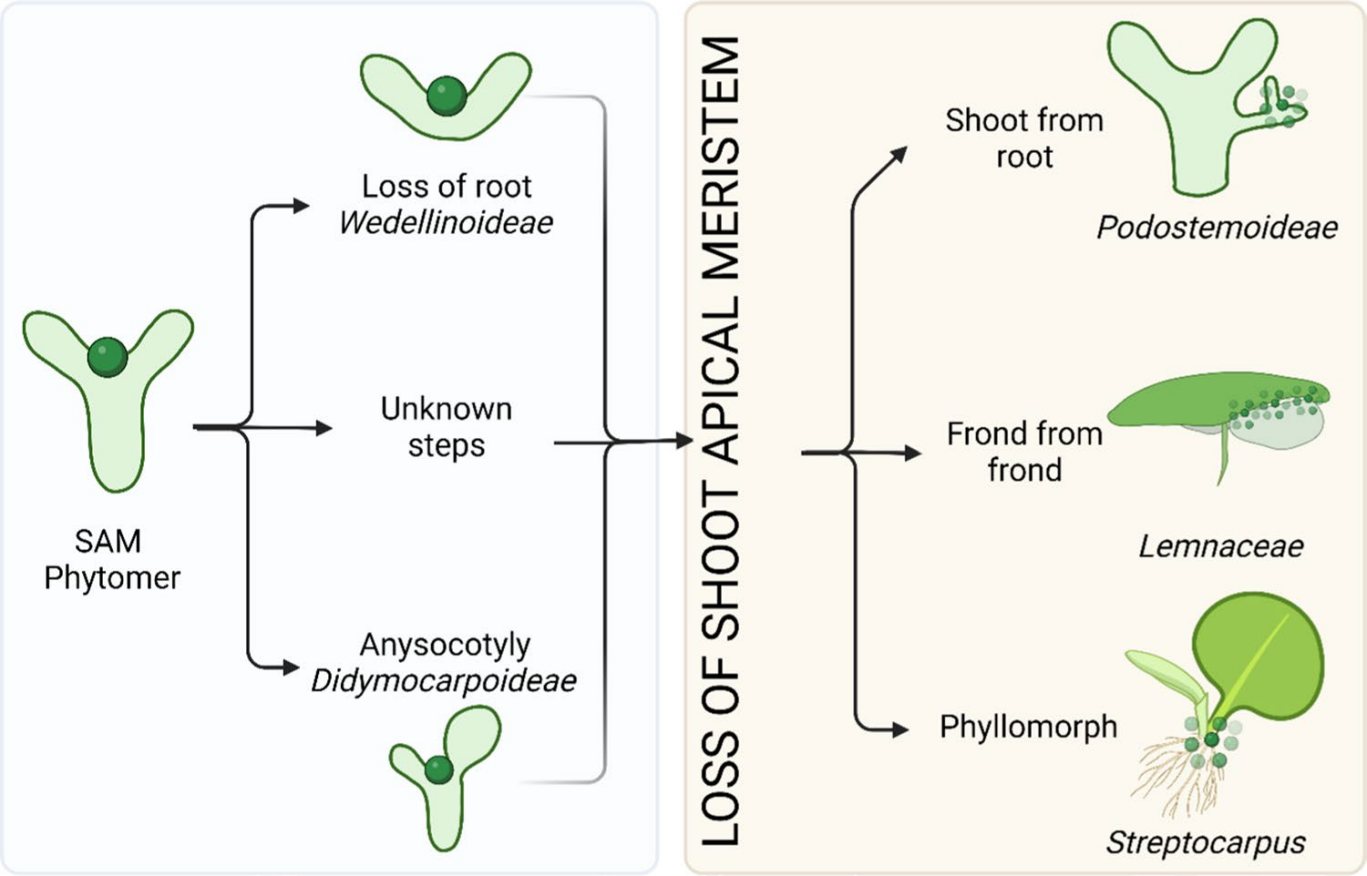
Schematic representation of the morphological differences between the three families considered, and the manifestation of SAM-like growth. Dark green sphere: SAM; small green spheres: regions of displacement of the meristematic activity

In this work, we consider several alterations of plant development, such as the alteration of the vegetative morphology: extreme morphological reduction and modification, absence of the phytomeric structure, substituted by a thallus (flattened, undifferentiated plant body), a phyllomorph, or a frond. We also consider the alteration of the vegetative reproduction: turion and frond formation; leaf development without SAM, leading to the formation of fronds, anisocotyly, and phyllomorphs (Table 1) (Figs. 2 and 7). Moreover, we report the expression of meristem specific genes during embryogenesis and plant body development, in the species analyzed (Table 2).

**Table 1:** Summary of morphological and developmental characteristics of the families considered in this work

Family	Embryogenic SAM	Growth module	Position of meristematic tissue	Shoot origin
Model plant	Yes	Phytomer	SAM and Axillary meristem	SAM and AM
Lemnaceae	No	Frond	At the base of the mother frond	At the base of the frond
Podostemaceae	No, in some Podostemoideae species	Thallus	At the base of the developing leaf	Determinate adventitious shoot from root
Gesneriaceae	No, in some Didymocarpoideae species	Phyllomorph	Three meristems, BM, PM, GM	GM

See text for details

**Table 2:** Summary of genes expression patterns and morphogenesis during both embryogenesis and seedling development of the families considered in this work

Morphology—embryogenesis							
		8 cells (proembryo)	16 cells	Late globular	Transition	Late heart	Mature embryo
*Arabidopsis thaliana*	Epigean tissues from layered SAMs	Apical embryo domain; L1 from epidermal precursor		OC formation	Central meristem; cotyledonary primordia from peripheral region	Periclinal divisions originate L2 and L3	Three-layered shoot meristem
Podostemaceae							
Podostomoideae *Z. lichenoides*	Determinate shoot between cotyledons		OC precursors	OC formation	L1 layer of a shoot meristem is not formed		No SAM
*H. japonicum*	No SAM	8 celled embryo uncorrected cell division; no L1 and OC precursor			No periclinal cells division and L2 and L3 formation		No SAM
Gesneriaceae							
Didymocarpoideae *S. rexii*				Embryogenesis histologically comparable that of Arabidopsis			No SAM
*Monophyllaea spp.*	No data available						
Lemnaceae	No data available						

Morphology—germination and development	
*Arabidopsis thaliana*	Two equal cotyledons; indeterminate SAM producing phytomers
Gesneriaceae	
Didymocarpoideae	Anysocotyly; no SAM; three meristems (BM, PM and GM) producing phyllomorphy and inflorescence; unifoliate and rosulate
Podostemaceae	
General	Dorsiventrally flattened photosynthetic body; if present, radicle and plumule are short-lived; outgrowth of the hypocotyl, leading to adventitious roots and shoots
*Tristichoidea*	SAM is present; buds develop in *ramuli*; shoot may be crustose (foliose); leaves derive from fusion of various shoot axes; adventitious shoots originate from roots
Weddellinoideae	Primary shoot between the cotyledons; adventitious shoots originate from roots
Podostemoideae	Mostly no SAM; plumular leaves (*Zeylanidium*) from embryonic shoot; adventitious root from hypocotyl; adventitious shoots from roots, without SAM
Lemnaceae	
General	The plant body is a single unit called a frond; new fronds arise from a preexisting one
Lemnoideae*; Spirodela*	The shoot consists of a frond, a prophyll, and roots; budding of subsequent fronds from two pockets
Lemnoideae*; Landoltia*	The shoot consists of a frond and a prophyll; budding of subsequent fronds from two pockets
Lemnoideae*; Lemna*	The shoot consists of a frond; budding of subsequent fronds from two pockets
Wolffioideae *Wolffiella, Wolffia*	No root; budding of additional frond from single pocket

Gene expression during embryogenesis						
Species	8 cells (proembryo)	16 cells	Late globular	Transition	Late heart	Mature embryo
*Arabidopsis thaliana*	WUS in putative OC precursor cells		WUS in shoot meristem primordium		WUS in OC; STM in shoot meristem	
Podostemaceae						
Podostemeoideae *Z. lychenoides*	STM in putative OC precursor cells		STM in the protodermal cells of the apical region	STM in putative OC precursor cells and at the cotyledon’ junction cells	Weak expression in cells between the cotyledon’s cells and in the junction cells	
Gesneriaceae						
Didymocarpoideae *S. rexii*	STM and WUS diffusely expressed in the embryo			STM in cotyledons and root apical meristem	No STM between the cotyledons	No STM; no WUS between cotyledons

Gene expression during plant development					
	STM	WUS	ARP	BP	CUC
*Arabidopsis thaliana*	SAM; ARP controls STM in leaves primordia	OC; CLV–WUS interaction maintains the stem cells	In primordia, but not in the meristem	In SAM during vegetative growth; downregulated in leave primordia	shoot organ boundary
Gesneriaceae					
Didymocarpoideae					
*S. rexii*	Isocotyly stage: at the base of cotyledons; anysocotyledony stage: at the base of macrocotyledon In phyllomorphs in PM, BM, GM	Isocotyly stage: at the base of cotyledons but not between them; anysocotyledony stage: at the base of macrocotyledon In phyllomorphs in PM, BM, GM	Macrocotyledon stage in BM; in phyllomorph in GM and lamina	Overlap with ARP expression	
*Monophyllea glabra*	GM		BM		
Podostemaceae					
Tristichoideae *Terniopsis minor*	Root, at the new shoot origin; absent in the leaf primordium	Root, at the new shoot origin; absent in the leaf primordium			
*Dalzella ubonensis* *Indodalzellia gracilis*	SAM, absent in the leaf primordium	SAM, absent in the leaf primordium			
Podostomideae *H. japonicum*	Root borne leaf primordium Leaf primordium Leaf basal part No in adult leaf	In the center of the first leaf primordium	Distal part of the developing vegetative leaf primordia and complements STM		
*Cladopus doianus*	Base of young leaf-shoots Young leaves primordia No in adult leaves				
*Zeylanidium tailichenoides*	At the bases of the cotyledon originating the first shoot. After, at the initiation site of the second shoot. Later in the whole primordium of the first shoot				At the base of cotyledon originating the first shoot At the initiation site of the new shoots

See text for details

In the families considered, the SAM is altered or absent and it does not arise during embryogenesis. In nature, the lack or modification of the SAM is not rare. For example, it is encountered in mosses (Bryophyta), liverworts (Marchantiophyta), and hornworts (Anthocerotophyta) (Mishler and Churchill 1984). In such species, growth and development are primarily controlled by the activity of intercalary or lateral meristems. In the Crassulaceae family, genus *Kalanchoe*, *Graptopetalum*, and *Crassula,* several species reproduce vegetatively by forming plantlets on the leaf margins in the absence of an SAM (Guo et al. 2015). Such plantlet formation requires the somatic initiation of organogenic and embryogenic programs present and active in leaves (Garces et al. 2007, 2014). Similarly, some species in the Poaceae show pseudovivipary, that is leafy shoot plantlets deriving from the conversion of the spikelet or part of it (Vega and Rúgolo de Agrasar 2006). Moreover, in Meliaceae*,* genus *Guarea* and *Chisocheton*, leaves can develop indeterminately as a result of meristematic activity of a leaf apical meristem which is very similar to the SAM (Fukuda et al. 2003). Genes known to be involved in the formation and maintenance of the SAM are involved in these types of processes (Jacome-Blasquez and Kim 2023).

The absence of SAM might have evolutionary relevance as part of an adaptive strategy to specific environmental conditions. In the Podostemaceae*,* the driving force could be an adaptation to fast water currents habitats (Rutishauser 2016; Kato et al. 2022). In fact, the flattened thallus-like structures minimize resistance to the water flow and the lack of a complex shoot system reduces the risk of damage from the current. Similarly, a vertical type of growth should have been counter selected also in the free-floating aquatic Lemnaceae (Ziegler et al. 2023). The degeneration of the root structure in duckweed is also probably the result of the adaptation to the environment, together with the modification of the function of their stomata, and the decrease in lignocellulose content (Ziegler et al. 2023). The aquatic and carnivorous plant *Utricularia gibba* (Lentibulariaceae) lacks roots and, probably as an adaptation to the aquatic environment, there is no clear distinction between leaves and stems (Chormanski and Richards 2012).

The absence of an SAM is a likely advantage also in environments where growing space is limited or crowded, such as in densely shaded areas; it allows the plants to adopt a more compact growth habit to efficiently utilize available resources and compete for light, water, and nutrients (Möller and Cronk 2001). The occurrence and maintenance of SAM absence in mutants during evolution may be a case of saltational evolution (Rutishauser 1995, 1997). In this view, the Podostemaceae mutants became stabilized and led to new taxa, while sister taxa remain relatively unchanged. A similar case is found in the extreme floral polymery in *Schefflera subintegra* (Araliaceae) (Nuraliev et al. 2014). Even if the absence of SAM and phytomers is observed in all three considered families, each one of them may have been subjected to a different evolutionary process, including diversification and specialization, genome evolution, and growth modularity. For example, in the unifoliate *Monophyllaea glabra*, *CUC* and *STM* are co-expressed during SAM initiation and the vegetative phase, thus extending the pre-mature SAM stage and resulting in neotenic meristems, which could represent a novel plant trait (Nakamura et al. 2024). Comparative analyses suggest some possible driving forces of the noted morphological alterations (Table 1). For example, anisocotyly is apparently a prerequisite for the evolution of the phyllomorph and may have occurred several times independently in different environments (Nishii et al. 2017).

### Comparative genetics and genomics

The molecular biology of the development of some organs of the species we considered allows to draw some general conclusions. Root formation requires the activity of genes which are shared with the other species; for example, *Spirodela* has the same root-supportive genes as rice (An et al. 2019). The formation of the turions in *S. polyrhiza* is triggered by a class of trancription factors known as Growth-Regulating Factors (GRFs) and hormones (Li et al. 2022). This is true also for other aquatic plants, such as *Potamogeton octandru* (He et al. 2018), where the number of GRFs increased during species differentiation. The floating nature of aquatic plants may support an evolutionary reduction of gene copies for lignin biosynthesis (Park et al. 2021) and of root-related genes, as observed in *Utricularia gibba* and in *U. vulgaris* (Barta et al. 2015). A further feature of aquatic plants is that they do not show a time-of-day gene expression pattern, as seen in land plants. Probably, this is due to the constant availability, for them, of nutrients present in water (Michael et al. 2017). In *Wolffia*, the decrease in circadian, light, and flowering time genes could be linked to the genome innovations responsible for the change in body plan (Michael et al. 2017).

It is well known that key genes like *STM* and *WUS*, if ectopically expressed, can revert differentiation and activate cell divisions leading to SAM and shoot and/or root formation (Gallois et al. 2002; Rashid et al. 2007; Negin et al. 2017). In spontaneous mutants where *KNOX* genes are ectopically expressed, novel structures may develop in non-conventional places (Golz et al. 2002). For example, in *Antirrhinum majus,* the *KNOX* novel overexpression results in the duplication of the petal tube to form a structure that resembles the spur of other Antirrhineae, while in barley, ectopic expression of the *Knox3* gene causes the appearance on the lemma of an extra flower with inverse polarity (Muller et al. 1995).

In the species, we have considered in this paper that the expression pattern of developmentally fundamental genes (and their interactions) in meristematic-like tissues is similar to what has been observed in the SAM of model angiosperms. In other words, morphological novelty may not be due to a novel set of gene expression, but rather to a modified balance among gene members of morphological regulatory networks (Nishii et al. 2010). This leads to fundamental questions: how biological forms do develop and what is the basis for their diversity. The unconventional *bauplan* of the three families we highlighted can help in addressing those questions and conceptualize how the balance of conservation versus divergence in gene regulatory networks yields different morphologies.

When it comes to genome size, a strong relationship between size and morphological complexity has been observed (An et al. 2018). Ancient allopolyploidization events have been associated to the rapid radiation of core Didymocarpinae (Xue et al. 2020b). Similarly, allopolyploidy is at the basis of adaptive radiation in the Caryophyllaceae family, genus *Schiedea*, probably because of differences in gene expression and/or alterations in coding sequences (Kapralov et al. 2013). Comparative genomics has shown that the duckweed genome contains a relatively low gene number and reduced gene families, a situation which may explain its highly reduced morphology. This is common with the other species, like the leafless parasitic *Cuscuta australis* (Yoshida and Kee 2021). Many of these gene loss events likely result from their parasitic lifestyle and massive changes of the body plan (Sun et al. 2018). Also, *Utricularia gibba* (Lentibulariaceae) experiences gene and genome size reduction (Veleba et al. 2014)*.* In this species, the *WOX* gene family experienced a differential expansion and contraction, where *WOX1* paralogs expanded and *WOX5* was lost. The increase of *WOX1* could correlate with leaf blade outgrowth and margin modification during the formation of the traps, i.e., the modified leaves that trap and digest preys; the loss of *WOX5* may likely be related to the lack of roots in the species (Carretero-Paulet et al. 2015). In the Lemnaceae, some of the orthologs in the *Argonaute* family are missing, probably as a consequence of their clonal growth habit (in fact, the missing genes are highly expressed, in Arabidopsis, in the pollen and seeds; Liu et al. 2009). Similarly, *WOX4* is absent (Ernst et al. 2023). *WOX4* regulates cell division in the procambium (Ohmori et al. 2013), and thus, its absence in the Lemnaceae may contribute to the observed simplification of vasculature.

A reduction of the genome size characterizes invasive species (Lavergne et al. 2010). Smaller size increases plant invasive potential leading to a higher early growth rate. This phenomenon may provide a selective advantage during the invasion process as a result of reduced resource allocation for the replication and maintenance of the DNA machinery; a faster cell cycle and a slightly higher mutation rate (Lavergne et al. 2010). This trend is evident in aquatic plants.

An increase of ancient mutation rates is evident in Podostemaceae, at first when the family originated and subsequently when the Podostemoideae diversified (Katayama et al. 2019, 2022). The variable exposure to UV light in open river areas may account for this phenomenon. Additionally, in the Podostemoideae, there are a greater number of cells which are actively dividing, somehow replacing the lacking SAM. Consequently, the number of mutations is higher than in model plants. Finally, organisms living in harsh environments might have evolved a higher tolerance for DNA damage, as a result of an evolutionary trade-off: tolerate some damage and risk mutations, or meticulously repair everything and potentially struggle to survive (Katayama et al. 2022).

The genomes of several of the species that we considered (i.e., *S. polyrhiza*, *W. Australiana*, and *L. minuta*) show the evidence of ancient whole-genome duplication, WGDs (Abramson et al. 2022; Xue et al. 2020a). The likely subsequent gene loss and genome readjustments may have contributed to the evolution of sets of genes specifically controlling fast growth rate and specific plant morphology (Qiao et al. 2022). WGDs, along with species switching to different environments, might have originated the ‘hopeful monsters’ at the base of their saltational evolution (Katayama et al. 2022). Further, specific gene families were lost only in *Zostera* and *Spirodela* (Wang and Messing 2015)*.* Not surprisingly for aquatic species, the genes lost were necessary to control stomatal gas exchange or to cope with UV radiation (Olsen et al. 2016). Other lost gene families include those controlling precursors involved in the perception of gravity and resistance to drought (Mizutani and Ohta 2010). Some other gene families had a quick expansion, like those involved in the response to hypoxia (Faizullah et al. 2021). The Nucleotide Leucine-Rich Repeats Receptors (NLR) genes and those for immune signaling are absent in aquatic species (for example in *S. polyrhiza*, *Z. marina*, and *Utricularia gibba)* (Baggs et al. 2020). Not all genes of those classes are equally lost: for example, *EDS1/PAD4* are absent only in species with a low number of NLR genes (Yang et al. 2022). The immune system of aquatic plants needs to be constitutively alerted, because these species are always in contact with pathogens: for this, they have a reduced silencing system of the repeat sequences, like retrotransposons and repeat disease-resistance genes (Baggs et al. 2019). Epigenetic reprogramming may also be involved in the establishment of the morphological features of the species considered. For example, the Lemnaceae are missing some genes involved in gene silencing, as a mechanism of adaptation to clonal propagation in the case of absence of methylation reprogramming (Ernst et al. 2023).

## Conclusions

The loss or reduction of the SAM in certain plant lineages has likely been driven by a combination of selective pressures and evolutionary constraints. Understanding the evolution and function of the SAM in diverse plant lineages can provide insights into the mechanisms of plant growth and development, as well as factors that have shaped plant diversity over millions of years of evolution. Morphological changes are often linked to or caused by changes in the expression of important regulatory genes or by variations in gene relationships within a regulatory network. The availability of sequences and expression data from an increasing number of non-model plants allows us to understand the evolution of transcriptional regulatory mechanisms as drivers of morphology changes in those “freak of nature” families (Das Gupta and Tsiantis 2018). Our review highlights a fascinating evolutionary strategy: the transfer of ancestral functions to new anatomical locations. Unlike typical plants where new leaves and shoots arise from the SAM, these unique lineages utilize alternative organs, like cotyledons or roots, for this purpose. This phenomenon, termed heterotopy, exemplifies a broader concept—heterochrony, which includes changes in gene expression and altered developmental timing of specific structures relative to others (Lacroix et al. 2005). Angiosperms, with their modular developmental program, are particularly prone to such functional transfers. Their "genetic subroutines" exhibit remarkable tolerance for repositioning. For instance, “leaf” modules are repeatedly deployed across various developmental stages and locations—cotyledons, juvenile and reproductive leaves, bracts, stamens, petals, and carpels.

These modules are subject to evolutionary forces leading to novel morphologies, potentially involving a functional shift to better adapt to specific ecological niches. Examples include light interception by cotyledons in *Streptocarpus*, or the complete suppression of vertical growth in Podostemaceae and Lemnaceae (Fig. 7).

## Data Availability

Not applicable.
